# Progranulin Is a Survival Factor for Axotomized Retinal Ganglion Cells in Adult Mice

**DOI:** 10.3390/cells15110988

**Published:** 2026-05-28

**Authors:** Lynn Michelle Grodzki, Stefanie Schlichting, Yue Hu, Sabine Helbing, Udo Bartsch

**Affiliations:** Department of Ophthalmology, Experimental Ophthalmology, University Medical Center Hamburg-Eppendorf, 20246 Hamburg, Germany; lynn.schau94@gmail.com (L.M.G.); st.schlichting@uke.de (S.S.); mia.huy@outlook.com (Y.H.); s.helbing@uke.de (S.H.)

**Keywords:** axonal regeneration, lentiviral vector, neural stem cells, neuroprotection, optic nerve, optic neuropathies, progranulin, retina, retinal ganglion cells

## Abstract

Progranulin (PGRN) is a secreted protein composed of 7.5 granulin domains. The protein is implicated in various functions, including cell survival, inflammation, lysosomal homeostasis, tumorigenesis, and aging. Haploinsufficiency and complete loss of PGRN function cause the neurodegenerative disorders frontotemporal lobar degeneration and neuronal ceroid lipofuscinosis type 11, respectively. In the nervous system, administration of exogenous PGRN has been shown to promote the survival of various nerve cell types under different pathological conditions and to stimulate neurite outgrowth in vitro and axonal regeneration in vivo. In the retina, PGRN dysfunction results in photoreceptor and retinal ganglion cell (RGC) loss, whereas PGRN administration promotes photoreceptor cell survival. In the present study, we analyzed whether a sustained intravitreal administration of PGRN promotes the survival of axotomized RGCs and the regrowth of the lesioned axons. To this end, we generated a PGRN-overexpressing clonal neural stem cell line and injected the cells into the vitreous cavity of a mouse optic nerve crush model. The progression of the lesion-induced degeneration of RGCs was studied at different time points after the nerve crush. The regeneration of the injured RGC axons into the distal optic nerve stump was analyzed one month after nerve lesioning. We found that the intravitreally administered PGRN slowed the degeneration of the injured RGCs for up to four months, the latest post-lesion interval analyzed. Furthermore, PGRN stimulated the regeneration of some RGC axons over long distances into the distal optic nerve stumps. Taken together, our results identify PGRN as a novel neurotrophic factor for retinal ganglion cells.

## 1. Introduction

Optic neuropathies, such as glaucoma, are a heterogeneous group of neurodegenerative diseases characterized by damage to the optic nerve and loss of retinal ganglion cells (RGCs), ultimately leading to progressive visual impairment and, eventually, blindness. The underlying pathomechanisms are not yet fully elucidated, which complicates the development of effective treatments for these conditions [1,2,3]. Elevated intraocular pressure (IOP) is considered the most important risk factor for glaucomatous damage, and pharmacological or surgical reduction in IOP is the only clinically proven treatment for the disease [4,5,6]. However, this approach is often only partially effective or fails in a substantial proportion of glaucoma patients. In addition, a considerable proportion of patients develop glaucoma despite having a normal IOP, indicating that IOP-independent mechanisms are involved in disease progression [4,7,8]. Due to these limitations, current research efforts are focusing on alternative treatment options, such as neuroprotective strategies that aim to slow or halt RGC degeneration and, in the case of traumatic optic nerve injuries, promote the regeneration of injured RGC axons [9,10,11,12,13]. This work has identified several neurotrophic factors (NTFs) that effectively protect RGCs from cell death in animal models of different optic neuropathies, including brain-derived neurotrophic factor (BDNF), ciliary neurotrophic factor (CNTF), and glial cell line-derived neurotrophic factor (GDNF) [14,15,16,17,18,19,20,21]. Furthermore, certain neuroprotective factors, such as CNTF and interleukin-6 (IL-6), have been shown to stimulate long-distance regeneration of lesioned RGC axons [22,23,24,25].

Progranulin (PGRN), also known as granulin-epithelin precursor, proepithelin, acrogranin, prostate cancer cell-derived growth factor, or epithelial transforming growth factor, is encoded by the *GRN* gene located on chromosome 17q21. PGRN is a secreted glycoprotein with a molecular mass of approximately 90 kDa that can be proteolytically cleaved into seven highly conserved cysteine-rich 6 kDa peptides designated granulins (GRN A through GRN G) and a 3.5 kDa peptide designated paragranulin [26,27,28,29]. In the central nervous system (CNS), PGRN is predominantly expressed in neurons and microglia cells [30,31,32]. PGRN is a multifunctional protein implicated in diverse biological processes, including cell survival, axonal outgrowth, embryogenesis, inflammation, lysosomal homeostasis, tumorigenesis, and aging [27,33,34,35]. Mutations in *GRN* that result in PGRN haploinsufficiency cause frontotemporal lobar degeneration (FTLD) with aberrant accumulation of transactivation response DNA-binding protein of 43 kDa (TDP-43), a neurodegenerative disease that manifests in adulthood and is characterized by neuronal loss predominantly in the frontal and temporal lobes [36,37]. Complete loss-of-function mutations in *GRN*, in comparison, cause the rare neurodegenerative lysosomal storage disorder neuronal ceroid lipofuscinosis type 11 (CLN11) [38,39], indicating a critical role of the protein in regulating lysosomal function.

Several studies have demonstrated that PGRN functions as a neurotrophic factor. While PGRN deficiency results in increased cell death upon exposure to cellular stress and in decreased neurite outgrowth of cultured neurons, administration or overexpression of PGRN has been shown to promote cell survival and neurite outgrowth of various nerve cell types, including cortical, motor, hippocampal, dorsal root ganglion, and trigeminal ganglion neurons [40,41,42,43,44,45,46,47,48,49,50]. In line with these in vitro observations, PGRN deficiency has been shown to exacerbate neurodegeneration and tissue damage and to slow axonal regeneration following acute insults to the CNS or peripheral nervous system (PNS) [50,51,52,53,54,55,56]. Conversely, administration of recombinant PGRN or PGRN overexpression has been demonstrated to attenuate tissue damage and to promote neuronal survival and axonal regeneration in animal models of various neurological disorders [41,57,58,59,60,61,62,63,64].

Of interest in the present context, PGRN also impacts the survival of retinal cell types. Intravitreal injections of recombinant PGRN have been demonstrated to promote the survival of photoreceptor cells in a mouse model of light-induced retinal degeneration [65] and a mouse model of retinal hypoxia [66]. Furthermore, mutations in the *GRN* gene cause progressive retinal degeneration and vision loss. The retinas of FTLD patients show significant thinning of the nerve fiber layer and ganglion cell complex and a reduction in macular volume [67], and CLN11 patients and CLN11 animal models exhibit progressive degeneration of photoreceptor cells and RGCs [38,67,68,69,70,71,72,73], demonstrating the importance of PGRN for retinal integrity.

Based on the observations that PGRN promotes nerve cell survival and axonal regeneration and that PGRN deficiency leads to degeneration of RGCs, the present study aimed to test whether intravitreally administered PGRN rescues RGCs from lesion-induced cell death. We also investigated whether PGRN stimulates long-distance regrowth of the injured RGC axons. To address these questions, we generated a clonal neural stem cell (NSC) line that stably overexpressed full-length murine PGRN and transplanted the modified NSCs into the vitreous cavity of a mouse optic nerve crush model. The effect of intravitreally administered PGRN on the survival of the injured RGCs was analyzed for up to four months following the lesion, while the extent of axonal regeneration in PGRN-treated animals was studied one month after nerve lesioning.

## 2. Materials and Methods

Intraorbital optic nerve lesions, intravitreal NSC transplantations and anterograde axonal tracing experiments were performed on adult (at least 2-month-old) C57BL/6J wild-type mice of both sexes. In some experiments, retinas from adult C57BL/6J mice that neither underwent an optic nerve lesion nor an intravitreal cell transplant were analyzed as a reference. NSC cultures were established from the cerebral cortices of 14-day-old C57BL/6J mouse embryos, as previously described [74,75]. The animals were obtained from the Animal Facility of the University Medical Center Hamburg-Eppendorf. All animal experiments were approved by the University and State of Hamburg Animal Care Committees and were conducted in accordance with the European Union Directive on animal experimentation 2010/63/EU.

In order to generate a clonal NSC line that overexpresses PGRN (hereafter referred to as PGRN-NSCs), the cells were transduced by spinoculation with a polycistronic lentiviral vector [76] encoding murine PGRN, the fluorescent reporter protein Venus, and a zeocin resistance under the control of a cytomegalovirus enhancer/chicken β-actin (CAG) promoter. For control experiments, cells were transduced with an identical vector but lacking the PGRN cDNA (hereafter referred to as control-NSCs). To establish clonal NSC lines with high transgene expression levels, the cells were subjected to several rounds of transduction. After each transduction, cells exhibiting the highest reporter gene expression were isolated using fluorescence-activated cell sorting and clonally expanded. Immunocytochemistry was used to analyze the expression of PGRN in undifferentiated PGRN- and control-NSC clones. Following fixation in phosphate-buffered saline (PBS) containing 4% paraformaldehyde (PFA) and blocking in PBS containing 0.3% Triton X-100 and 0.1% bovine serum albumin (BSA; both from Sigma-Aldrich, St. Louis, MO, USA), cells were incubated with rat anti-mouse PGRN antibodies (1:1000; R&D Systems GmbH, Wiesbaden, Germany). Primary antibodies were detected with Cy3-conjugated donkey anti-rat secondary antibodies (1:200; Dianova, Hamburg, Germany), and cell nuclei were stained with 4′,6′-diamidino-2-phenylindole (DAPI; Sigma-Aldrich). To estimate the amount of secreted PGRN, 5 × 10^5^ PGRN-NSCs or control-NSCs from a low and a high passage were cultivated in 500 μL of serum-free culture medium for 24 h, and 30 µL of the conditioned culture supernatants were then subjected to Western blot analysis. The samples were separated using 10% SDS-PAGE, and the proteins were transferred to nitrocellulose membranes. Non-specific binding was blocked by incubating the membrane in Intercept (TBS) Protein-Free Blocking Buffer (LI-COR Biosciences, Lincoln, NE, USA) for one hour at room temperature. The membranes were incubated with rat anti-mouse PGRN antibodies (1:250; R&D Systems GmbH) overnight at 4 °C, followed by incubation with an IRDye 800CW-conjugated anti-rat secondary antibody (1:20,000; LI-COR Biosciences) at room temperature for 1 h. The amount of PGRN in supernatant from the PGRN-NSC clone at different passages was estimated by densitometric analysis of the immunoreactive bands using a serial dilution of recombinant mouse PGRN (R&D Systems GmbH) as a reference. Analysis of the blots was performed using a LI-COR Odyssey Imaging System and Empiria Studio^TM^ software 1.3.0.83 (LI-COR Biosciences). Experiments were performed in triplicate.

The animals were deeply anesthetized, and their optic nerves were crushed intraorbitally with watchmaker’s forceps, as previously described [77,78]. Criteria to include animals in the study included lack of retinal bleeding, intact lenses, and loss of the pupillary light reflex. One day after the optic nerve crush, 2 μL of vitreous fluid was removed from the eyes, and the same volume of PBS containing 8 × 10^5^ cells of either the control-NSC clone or the PGRN-NSC clone was slowly injected into the vitreous cavity. In some animals with grafted control- or PGRN-NSCs, anterograde axonal tracing experiments were performed 27 days after the optic nerve crush. After removal of 2 µL of vitreous fluid, the same volume of a saturated solution of biotin-N-hydroxysuccinimidester (Sigma-Aldrich) diluted 1:1 with dimethylformamide (Carl Roth GmbH + Co. KG, Karlsruhe, Germany) and ethanol was slowly injected into the vitreous cavity [78]. Following each procedure (i.e., optic nerve crush, intravitreal cell transplantation, or anterograde tracer injection), the eyes were treated with antibiotic eye drops (Oftaquix; Santen GmbH, Munich, Germany) and an eye gel containing wound-healing and moisturizing compounds (Corneregel; Bausch + Lomb Incorporated, Berlin, Germany).

The impact of the intravitreally grafted NSCs on the survival of the axotomized RGCs was analyzed 14, 28, 56, and 112 days post-lesion (dpl; *n* = 6 biological replicates for each treatment and post-lesion time point). The eyes were fixed in PBS containing 4% PFA for 15 min. Subsequently, the retinas were isolated and flat-mounted onto nitrocellulose membranes, fixed again in 4% PFA for one hour, and blocked in PBS containing 0.1% BSA and 1% Triton X-100. To visualize the surviving RGCs, retinal flatmounts were incubated overnight with rabbit antibodies directed against brain-specific homeobox/POU protein 3A (BRN-3A; 1:1000; Synaptic Systems, Göttingen, Germany), followed by Cy3-conjugated donkey anti-rabbit secondary antibodies (1:200; Dianova) and DAPI. An epifluorescence microscope (OLYMPUS IX51; Olympus, Hamburg, Germany) and imaging software (cellSens Entry 1.4; Olympus) were used to capture five consecutive micrographs from the optic disc to the periphery of each quadrant of the retinal flatmounts, covering a retinal area of approximately 2 mm^2^. A blinded observer counted the BRN-3A-positive RGCs using Photoshop CC software CC 2019 version 20.0.8 (Adobe Inc., San Jose, CA, USA). Representative micrographs of retinal flatmounts for qualitative documentation were taken in areas close to the optic disc using an AxioObserverZ.1 microscope equipped with an ApoTome.2 (Carl Zeiss AG; Oberkochen, Germany).

Immunohistochemical analysis on cryosections was performed on retinas from eyes with transplanted PGRN-NSCs (hereafter referred to as PGRN-treated retinas; *n* = 5) or control-NSCs (hereafter referred to as control retinas; *n* = 5) 14 days after the lesion. Eyes from mice that received neither an optic nerve crush nor an intravitreal cell transplant (hereafter referred to as untreated retinas; *n* = 5) were processed as a reference. After removal of part of the cornea, eyes were immersion-fixed in PBS containing 4% PFA overnight, cryoprotected in an ascending series of sucrose, frozen, sectioned at a thickness of 25 µm using a cryostat, and blocked in PBS containing 0.1% BSA and 0.3% Triton X-100. RGCs were stained with goat anti-human BRN-3A antibodies (1:200; Santa Cruz Biotechnology Inc., Santa Cruz, CA, USA) followed by Cy3-conjugated donkey anti-goat secondary antibodies (Dianova). To analyze the uptake of PGRN into the retina and surviving RGCs in PGRN-treated animals, sections were either incubated with sheep anti-human PGRN antibodies (1:500; R&D Systems GmbH) or simultaneously with anti-PGRN and rabbit anti-human β-tubulin III antibodies (1:20,000; Sigma Aldrich). Primary antibodies were detected with Cy2- or Cy3-conjugated secondary antibodies (Dianova). To enable direct comparison of the PGRN fluorescence intensity, immunostainings of PGRN-treated retinas, control retinas and untreated retinas were performed in parallel, and sections were imaged using identical microscope settings.

Axonal regeneration was assessed 28 days after the optic nerve crush in animals with intravitreally grafted control-NSCs or PGRN-NSCs (*n* = 9 for each treatment). The animals were euthanized, and the eyes with attached optic nerves were fixed in 4% PFA, cryoprotected, and embedded in Tissue-Tek (O.C.T. Compound; Sakura Finetek USA Inc., Torrance, CA, USA). Longitudinal optic nerve sections, 25 µm thick, were prepared using a cryostat (Leica CM1950; Leica Mikrosysteme Vertrieb GmbH, Wetzlar, Germany). The sections were incubated overnight at room temperature with Cy3-conjugated streptavidin (1:200; Dianova), stained with DAPI, and mounted onto slides. The three optic nerve sections containing the longest regrown axons distal to the lesion site were chosen from each animal for analysis. Zen 2.1 software (Carl Zeiss AG) was used to measure the length of the longest regrown axon in each animal.

Statistical analysis of data was performed using a two-way analysis of variance (ANOVA) test followed by a Bonferroni post hoc test. Differences were considered statistically significant at *p* < 0.05.

## 3. Results

### 3.1. Characterization of the Modified NSC Clones In Vitro

**Figure 1:**
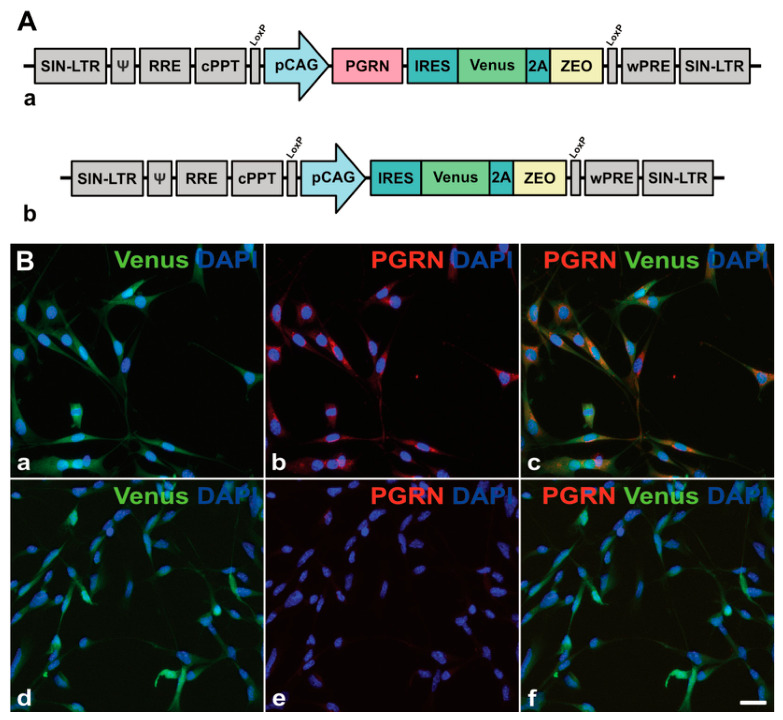
Expression of PGRN in the clonal NSC lines. (**A**) Schematic representation of the lentiviral vectors used to generate PGRN-NSCs (**Aa**) or control-NSCs (**Ab**). (**B**) Immunocytochemical analyses of the NSC lines revealed co-expression of PGRN and Venus in all cells of the PGRN-NSC clone (**Ba**–**Bc**). In contrast, control-NSCs expressed Venus but not PGRN (**Bd**–**Bf**). Abbreviations: Ψ, packaging signal; cPPT, central polypurine tract; DAPI, 4′,6-di-amidino-2-phenylindole; IRES, internal ribosome entry site; LoxP, recognition site of Cre recombinase; NSCs, neural stem cells; pCAG, cytomegalovirus enhancer/chicken β-actin promotor; PGRN, progranulin; RRE, rev-responsive element; SIN-LTR, self-inactivating long-terminal repeat; wPRE, woodchuck hepatitis virus posttranscriptional regulatory element; ZEO, zeocin. Scale bar: 25 μm.

A PGRN-overexpressing clonal NSC line was generated by transducing NSCs with a lentiviral vector encoding PGRN and the fluorescent reporter protein Venus under regulatory control of a CAG promoter (Figure 1(Aa)). NSCs for control experiments were transduced with a lentiviral vector encoding Venus but lacking the PGRN cDNA (Figure 1(Ab)). To augment the expression level of the transgenes in the clonal PGRN-NSC and control-NSC lines, cells were subjected to multiple rounds of transductions with the respective viral vectors. Each transduction was followed by selection of the cells with the highest Venus expression levels by fluorescence-activated cell sorting and subsequent clonal expansion. To evaluate the expression of PGRN in the cell lines used in this study, NSC cultures were immunostained with anti-PGRN antibodies. Experiments revealed that all cells in the PGRN-NSC line co-expressed PGRN and the fluorescent reporter protein Venus (Figure 1(Ba–Bc)). Control-NSCs stained in parallel were Venus-positive but PGRN-negative (Figure 1(Bd–Bf)).

**Figure 2:**
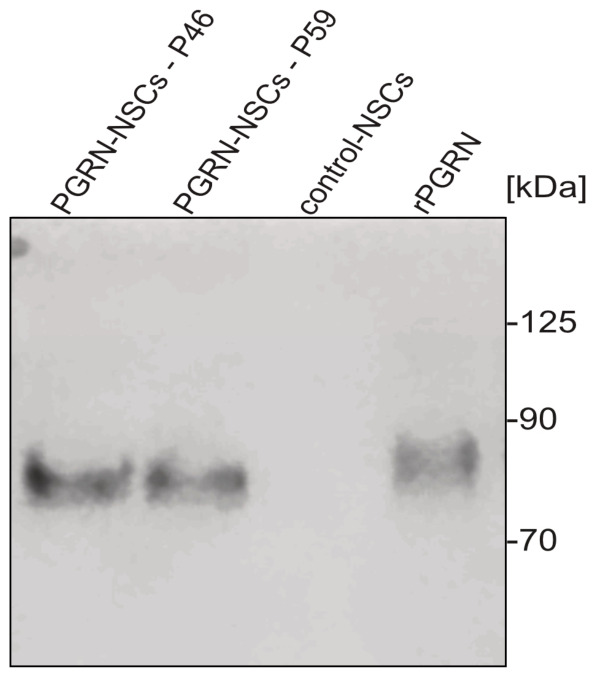
Immunoblot analysis of culture supernatants. Supernatants from the PGRN-NSC clone contained similar amounts of PGRN at passages 46 and 59. Supernatants from the control-NSC clone were PGRN-negative. Recombinant mouse PGRN was loaded as a reference. Abbreviations: kDA, kilodalton; NSCs, neural stem cells; P, passage; PGRN, progranulin; rPGRN, recombinant progranulin.

Further characterization of the cell lines was conducted using quantitative immunoblot analyses of cell culture supernatants. The results showed that PGRN was released from PGRN-NSCs into the supernatant (Figure 2). In comparison, supernatants from control-NSCs did not contain detectable amounts of PGRN (Figure 2). After cultivation of 5 × 10^5^ PGRN-NSCs in 500 μL culture medium for 24 h, quantitative Western blot analyses revealed the presence of 464 ± 35 ng PGRN (mean ± SEM, *n* = 3) in supernatants of the PGRN-NSC clone at passage 46. Supernatants of the same clonal cell line were analyzed after an additional 13 passages contained similar amounts of PGRN (383 ± 36 ng), indicating stable expression of the protein.

### 3.2. PGRN-NSCs Promote the Survival of Axotomized RGCs

**Figure 3:**
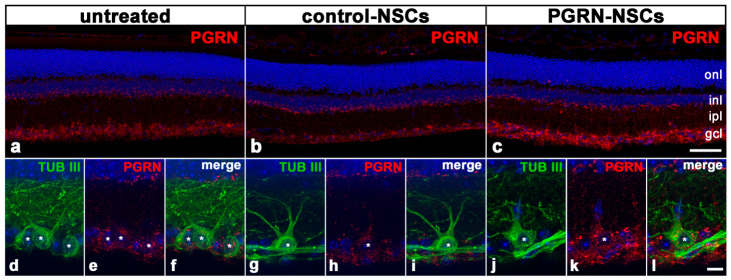
PGRN immunohistochemistry in PGRN-treated and control retinas. Immunostainings of retinas 14 dpl revealed significantly higher PGRN levels in PGRN-treated animals (**c**) than in untreated (**a**) or control (**b**) animals. Furthermore, PGRN levels in β-tubulin III-positive RGCs in PGRN-treated retinas 14 dpl (**j**–**l**) were significantly higher than in RGCs in untreated (**d**–**f**) or control (**g**–**i**) retinas. RGC nuclei in panels (**d**–**l**) are labeled with white asterisks. Abbreviations: dpl, days post-lesion; gcl, ganglion cell layer; inl, inner nuclear layer; ipl, inner plexiform layer; NSCs, neural stem cells; onl, outer nuclear layer; PGRN, progranulin; RGCs, retinal ganglion cells; TUB III, β-tubulin III. Scale bar in (**c**) for (**a**–**c**): 50 µm; and in panel (**l**), for panels (**d**–**l**): 10 µm.

To determine whether the intravitreally administered PGRN was taken up by the retinas, cryosections of PGRN-treated retinas and control retinas were stained 14 days after the lesion using anti-PGRN antibodies. Retinas from animals that had not undergone an optic nerve lesion or cell transplantation were processed in parallel for comparison. In untreated retinas, PGRN was predominantly expressed in the inner retina (i.e., the ganglion cell layer and the inner nuclear layer), with weaker expression in the outer retina (i.e., the outer nuclear layer; Figure 3a), in agreement with other reports [68,71,79]. Notably, we consistently observed a significantly stronger PGRN signal in PGRN-treated retinas than in control retinas or untreated retinas, particularly in the ganglion cell layer (Figure 3a–c). PGRN levels in the control retinas were slightly lower than in untreated retinas, possibly due to the loss of RGCs. Interestingly, we also observed significantly higher PGRN levels in the surviving β-tubulin III-positive RGCs in PGRN-treated retinas than in RGCs in control or untreated retinas (Figure 3d–l).

**Figure 4:**
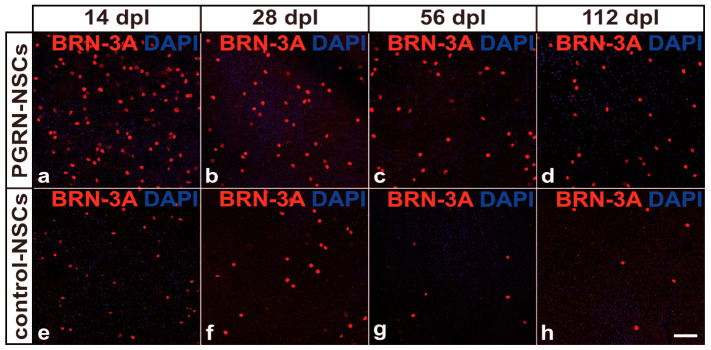
BRN-3A-labeled RGCs in flat-mounted retinas. Retinas treated with PGRN-NSCs (**a**–**d**) or control-NSCs (**e**–**h**) 14 (**a**,**e**), 28 (**b**,**f**), 56 (**c**,**g**), and 112 (**d**,**h**) days after an intraorbital optic nerve crush. PGRN-treated retinas contained significantly higher numbers of surviving RGCs than control retinas at all post-lesion time points. Abbreviations: BRN-3A, brain-specific homeobox/POU domain protein 3A; DAPI, 4′,6-di-amidino-2-phenylindole; dpl, days post lesion; NSCs, neural stem cells; PGRN, progranulin; RGCs, retinal ganglion cells. Scale bar: 50 μm.

**Figure 5:**
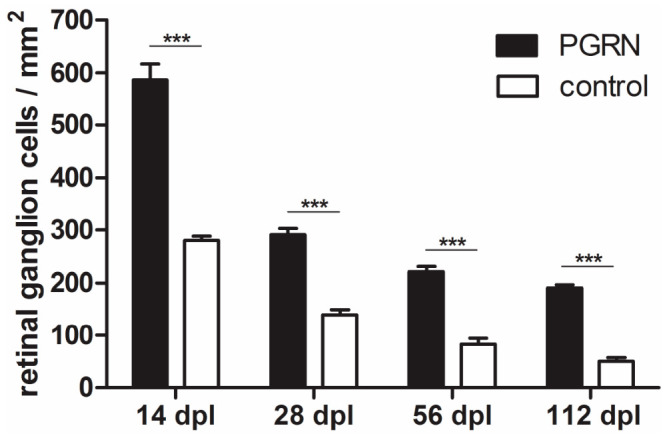
Quantitative analysis of RGC survival. The density of BRN-3A-positive RGCs in PGRN-treated retinas (filled bars) and control retinas (open bars) 14, 28, 56, and 112 days after an intraorbital optic nerve lesion. Each bar represents the mean value (±SEM) from six retinas. *** *p* < 0.001 according to a two-way ANOVA followed by a Bonferroni post hoc test. Abbreviations: dpl, days post lesion; PGRN, progranulin; RGCs, retinal ganglion cells.

To evaluate whether the exogenously administered PGRN has slowed the degeneration of the injured RGCs, the cells were visualized in flat-mounted retinas 14, 28, 56, and 112 days after the optic nerve lesion using antibodies against BRN-3A (Figure 4a–d). Flat-mounted retinas from animals treated with control-NSCs were evaluated as a reference (Figure 4e–h). Qualitative inspection of the retinal flatmounts revealed a significantly higher number of BRN-3A-positive ganglion cells in PGRN-treated eyes than in control eyes up to four months after the nerve lesion (Figure 4), the latest post-lesion time point analyzed. Improved survival of lesioned RGCs in PGRN-treated retinas compared to control retinas was also evident in retinal sections (Figure S1). Quantitative analyses confirmed that the PGRN treatment had slowed the lesion-induced loss of RGCs. Retinal flatmounts from eyes that had received injections of the PGRN-NSC clone contained 586.3 ± 30.5 RGCs/mm^2^ (mean ± SEM) 14 dpl, 291.0 ± 12.2 RGCs/mm^2^ 28 dpl, 220.8 ± 9.8 RGCs/mm^2^ 56 dpl, and 190.2 ± 6.9 RGCs/mm^2^ 112 dpl (*n* = 6 for each post-lesion interval; Figure 5). In comparison, RGC densities in animals with intravitreally grafted control-NSCs were significantly lower at all post-lesion time points, with 280.5 ± 8.2 RGCs/mm^2^ at 14 dpl, 155.2 ± 4.8 RGCs/mm^2^ at 28 dpl, 82.7 ± 11.3 RGCs/mm^2^ at 56 dpl, and 50.0 ± 6.6 RGCs/mm^2^ at 112 dpl (*n* = 6 for each post-lesion interval; Figure 5). Thus, retinas treated with PGRN-NSCs contained 2.1-, 1.9-, 2.7-, and 3.8-fold more surviving RGCs than retinas treated with control-NSCs 14, 28, 56, and 112 days after nerve lesioning, respectively. The difference in the number of RGCs between the PGRN-treated retinas and control retinas was statistically significant at all analyzed post-lesion time points (*p* < 0.001, two-way ANOVA followed by a Bonferroni post hoc test; Figure 5).

### 3.3. Axonal Regeneration

**Figure 6:**
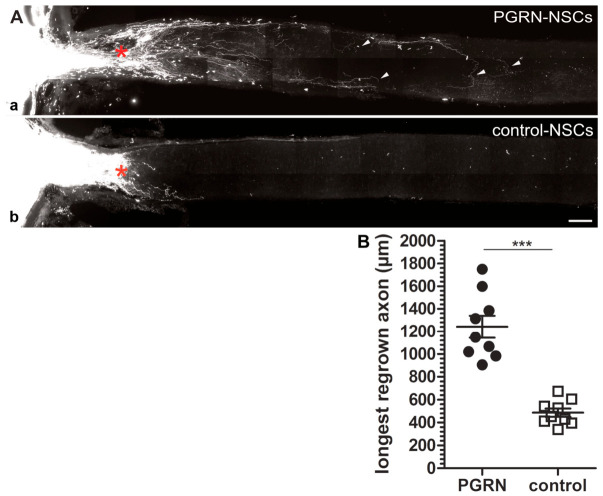
Regeneration of intraorbitally lesioned RGCs. (**A**) RGC axons were anterogradely labeled 28 days after an optic nerve crush in animals with grafted PGRN-NSCs (**Aa**) or control-NSCs (**Ab**). Note the irregular trajectories of the regrown axons in the PGRN-treated animals (**Aa**). Also note that some axons made U-turns (some are marked with arrowheads in (**Aa**)) Red asterisk in (**Aa**,**Ab**) indicates the lesion site. (**B**) The length of the longest regrown axon in the distal optic nerve stumps of PGRN-treated (filled circles) and control animals (open squares). *** *p* < 0.001 according to Student’s *t*-test. Abbreviations: NSCs, neural stem cells; PGRN, progranulin; RGCs, retinal ganglion cells. Scale bar: 100 μm.

PGRN has been shown to stimulate neurite outgrowth from various nerve cell types in vitro and to promote axonal regeneration in vivo. We therefore analyzed whether the intravitreally administered PGRN had stimulated the regrowth of the intraorbitally lesioned RGC axons across the lesion site into the distal optic nerve stumps. One month after the nerve lesion, some RGC axons in PGRN-treated animals had extended over considerable distances into the distal nerve stumps (Figure 6(Aa)). The mean length of the longest regrown axons in PGRN-treated animals was 1240 ± 97 μm (mean ± SEM; *n* = 9; *p* < 0.001; Figure 6B), whereas the mean length of the longest regrown axons in control animals was 487 ± 36 μm (mean ± SEM; *n* = 9). Axons in PGRN-treated animals exhibited tortuous trajectories, with some making U-turns and growing back towards the retina (Figure 6(Aa)), strongly suggesting that these axons were regenerated rather than spared by the nerve lesion. However, PGRN only stimulated a relatively small number of RGCs to regrow their axons over long distances beyond the crush site.

## 4. Discussion

PGRN is a secreted pleiotropic growth factor implicated in diverse functions, including tissue repair, cell survival, axon growth, and the regulation of inflammatory processes and lysosomal function [27,33,35]. In the CNS, PGRN is predominantly expressed in neurons and microglial cells [30,31,32]. Haploinsufficiency of PGRN causes FTLD [36,37], whereas complete loss of function causes the neurodegenerative lysosomal storage disorder CLN11 disease, a rare subtype of neuronal ceroid lipofuscinosis [38,39]. Dysregulated PGRN expression has also been associated with an increased risk of other neurodegenerative disorders such as Alzheimer’s disease, Parkinson’s disease, amyotrophic lateral sclerosis or limbic-predominant age-related TDP-43 encephalopathy [27,33,35], further demonstrating the importance of PGRN in the nervous system. Of note in the present context, the retinas of FTLD and CLN11 patients and *Grn* knock-out (ko) mice exhibit degeneration of RGCs [38,39,67,68,69,80]. Significant loss of RGCs in *Grn* ko mice was observed as early as postnatal day 9 [69], with only around 60% of RGCs remaining in 18-month-old mutants [68]. Based on these findings, the present study aimed to investigate the effects of exogenously administered PGRN on the survival and axonal regeneration of intraorbitally lesioned RGCs using a mouse optic nerve crush model.

Several in vitro studies have provided evidence that PGRN functions as a neurotrophic factor, promoting survival and neurite outgrowth of various nerve cell types. PGRN deficiency rendered neurons more vulnerable to cellular stress and significantly decreased neurite outgrowth, phenotypes that were reversed by PGRN overexpression or administration of recombinant PGRN [44,46,47]. PGRN also promoted cell survival and neurite outgrowth of diverse nerve cell types from the CNS and PNS of wild-type mice, including cortical, motor, hippocampal, trigeminal and dorsal root ganglion neurons [40,41,42,44,48,49,50,81]. These beneficial effects on cell survival and neurite outgrowth were also observed after administration of GRN E [40,42,43,44], indicating that the C-terminus of PGRN conferred the pro-survival and neurite outgrowth-promoting activity. The neurotrophic effects of extracellular PGRN have been linked to the activation of signaling pathways such as the mitogen-activated protein kinase/extracellular signal-regulated kinase (MAPK/ERK) or phosphatidylinositol-3-kinase (PI3K)/AKT pathway. However, the receptors initiating the signaling cascades remain elusive [46,48,49]. The C-terminus of PGRN binds to the neuronal receptor sortilin (SORT1) which regulates extracellular PGRN levels by mediating its endocytotic uptake and trafficking to the lysosomes [82,83]. However, interfering with PGRN–SORT1 interactions did not abolish PGRN’s neurotrophic effects [43,44]. Of note, a recent study indicates that binding of PGRN to cell surface signaling receptors is not necessary for promoting cell survival. Expression of L-PGRN, a lysosome-targeted PGRN variant that is not secreted but trafficked to the lysosomes where it is cleaved into granulins, protected cortical neurons more consistently from N-methyl-D-aspartate (NMDA) excitotoxicity than PGRN by attenuating the contribution of autophagy to excitotoxic cell death [45]. In the present study, we have used a cell-based intravitreal delivery strategy to administer PGRN continuously to the retina of a mouse optic nerve crush model. Notably, clinical trials have demonstrated the feasibility of translating cell-based intravitreal treatment strategies into clinical applications using the so-called encapsulated cell technology. Revakinagene taroretcel-lwey (also known as NT-501; Neurotech Pharmaceuticals, Cumberland, RI, USA) is a device containing immortalized retinal pigment epithelial cells genetically modified to secrete CNTF. Intravitreal implantations of the device into patients with retinitis pigmentosa, age-related macular degeneration, macular telangiectasia type 2 or glaucoma showed that it was well-tolerated and slowed retinal degeneration and eventually deterioration of retina function [84,85]. Remarkably, a recent analysis of explanted devices revealed that the encapsulated cells survived for more than 14 years in the vitreous cavity and secreted CNTF at a rate sufficient to promote photoreceptor survival [86].

Analysis of the PGRN-overexpressing clonal cell line used in this study revealed stable secretion of about 400 ng PGRN/5 × 10^5^ cells/24 h. In previous studies using NSC clones overexpressing other neurotrophic factors or the lysosomal protease cathepsin D, we have shown that the intravitreally injected cells stably expressed the transgenes and survived in the vitreous cavity of mouse models of retinitis pigmentosa [74], traumatic optic neuropathies [14,75] or neuronal ceroid lipofuscinosis [87,88] for up to eight months [25]. The transplanted NSCs stopped proliferating as assessed by Ki67 expression and did not integrate into the host retinas [74,75,87]. Consistent with these findings, we observed significantly higher PGRN levels in the inner retina and in the surviving RGCs in PGRN-treated animals than in control or untreated animals. Furthermore, the grafted PGRN-NSCs conferred long-term neuroprotective effects on injured RGCs (see below), and neither PGRN-NSCs nor control-NSCs integrated into the host retinas, as indicated by the absence of Venus-positive cells in retinal sections. Using antibodies to BRN-3A, a reliable marker for identifying and quantifying RGCs [89], we indeed found significantly more viable RGCs in eyes with injected PGRN-NSCs than in control eyes at all post-lesion time points examined (i.e., 14, 28, 56, and 112 dpl). Improved survival of RGCs in PGRN-treated retinas was also evident in retinal sections. Notably, four months after the lesion, retinas treated with PGRN-NSCs contained approximately 3.8-fold more BRN-3A-positive RGCs than retinas treated with control-NSCs. We have previously studied the neuroprotective effects of CNTF and GDNF in an optic nerve crush model, employing the same cell-based intravitreal delivery strategy. Both CNTF and GDNF have been shown to effectively promote RGC survival in animal models of various optic neuropathies [9,10,11]. Four months after the lesion, the RGC survival rates achieved with these factors were in the range of those observed in PGRN-treated retinas, with about 4.8- and 4.6-fold more RGCs in CNTF-treated and GDNF-treated retinas, respectively, than in control retinas [25,75]. These results indicate that PGRN is a potent neurotrophic factor for RGCs that significantly slows the lesion-induced degeneration of these neurons over a considerable period of time.

In the retina, PGRN deficiency not only causes loss of RGCs but also of photoreceptor cells [39,68,71,73,79]. Zin and colleagues studied the impact of an intravitreal and a systemic PGRN gene therapy on retinal degeneration in *Grn* ko mice, employing an AAV2.7m8 capsid capable of pan-retinal transduction and an AAV9.2YF capsid capable of crossing the blood–brain and blood–retina barriers, respectively [79]. Unexpectedly, treatment of adult *Grn* ko mice with the intravitreal gene therapy had either no effect on retina and photoreceptor layer thinning, or even accelerated retina thinning when aged mice were treated. Notably, however, treatment of young postnatal *Grn* ko mice with the systemic gene therapy slowed the thinning of the retina and the photoreceptor layer compared to controls [79]. Another study has shown that administration of recombinant PGRN protected the photoreceptor cell line 661W against H_2_O_2_- and light-induced cell death in a dose-dependent manner [65,90]. More importantly, and in analogy to the effects observed for injured RGCs, intravitreally administered recombinant PGRN rescued photoreceptor cells from light- or hypoxia-induced cell death in wild-type mice. Furthermore, the treatment attenuated deterioration of retinal function as assessed by electroretinography [65,66]. Taken together, the studies discussed above and the findings of the present study identify PGRN as a neuroprotective factor for the two major retinal nerve cell types, ganglion cells and photoreceptor cells.

The beneficial effects of PGRN on tissue integrity and neuronal survival have also been observed in other disease models. For instance, PGRN gene therapy rescued nigrostriatal neurons against 1-methyl-4-phenyl-1,2,3,6-tetrahydropyridine (MPTP) toxicity and preserved locomotor function in a mouse model of Parkinson’s disease, presumably by dampening the MPTP-induced inflammatory response [64]. Conversely, MPTP-induced neurodegeneration and neuroinflammation was exacerbated in *Grn* ko mice compared to wild-type mice [52]. The expression of the pro-inflammatory cytokines tumor necrosis factor-α, interleukin-1β and IL-6 was elevated while the expression of the anti-inflammatory cytokine IL-10 was decreased in activated PGRN-deficient microglia, and conditioned media from these cells promoted the cell death of co-cultured neurons. PGRN overexpression normalized the dysregulated cytokine expression in activated microglia cells [52]. Traumatic brain or spinal cord injury also aggravated tissue damage and neuron loss in *Grn* ko mice, and resulted in elevated expression of pro-inflammatory and decreased expression of anti-inflammatory mediators, increased lysosomal biogenesis in activated microglia, and augmented astrogliosis [54,55]. Administration of recombinant PGRN or neuron-restricted PGRN overexpression attenuated the exaggerated neurological phenotype of the mutant mice and mitigated the exacerbated injury-induced inflammatory response [53,56,91]. Of note, treatment with recombinant PGRN has been shown to attenuate neurodegeneration and promote recovery of motor functions in a rat spinal cord injury model, and these outcomes have been linked to a blunted neuroinflammatory response and to an enhanced autophagic flux in microglia [57]. Furthermore, administration or overexpression of PGRN reduced infarct volume, attenuated neurodegeneration and neurological deficits, and decreased mortality in animal models of ischemic and hemorrhagic stroke. The therapeutic outcomes of the PGRN treatments were also attributed to modulation of neuroinflammation [58,59,62], and to activation of pro-survival signaling pathways [60], and improved preservation of the blood–brain barrier [58,92]. Taken together, PGRN has been shown to promote neuronal survival under diverse pathological conditions, and may confer this pro-survival activity through multiple mechanisms.

Based on the finding that PGRN stimulates neurite outgrowth in vitro, we additionally performed anterograde tracing experiments to study whether PGRN promotes axonal regeneration of the injured RGCs. In the animals treated with PGRN, a few RGC axons extended over considerable distances across the lesion site into the distal optic nerve stumps. The average length of the longest regrown axons distal to the crush site in PGRN-treated mice was approximately 1200 µm, compared to around 500 µm in control animals. The traced axons in PGRN-treated animals followed irregular trajectories, and some made U-turns and grew back towards the retina, strongly suggesting that they correspond to regenerated axons rather than to axons that were spared by the lesion. However, the number of regenerated axons was low compared to the number of surviving RGCs. A striking discrepancy in the number of regenerating and surviving RGCs is a common finding regardless of the pro-regenerative treatment used, as reviewed in detail elsewhere [93,94,95]. Taken together, our results identify PGRN as a novel factor that stimulates long-distance regeneration of injured RGCs.

A role of PGRN in axonal regeneration was also found in other studies, which showed that PGRN deficiency slowed, while PGRN administration accelerated, the regrowth of damaged nerve fibers. For instance, facial nerve injury in *Grn* ko mice resulted in delayed axonal regeneration, as indicated by a delayed recovery of whisker movements, and this delay was fully rescued by the overexpression of human PGRN [51]. The authors also showed that the neurotrophic activity was mediated by the C-terminus of neuronally expressed PGRN, and that PGRN increased the enzymatic activity of the lysosomal protease cathepsin D (CTSD). Interestingly, functional recovery after facial nerve injury progressed normally in *Grn* or *Ctsd* heterozygous mice, but was delayed in *Grn*/*Ctsd* double heterozygous mice, suggesting that PGRN impacts axonal regeneration by improving lysosomal function. However, PGRN overexpression in wild-type mice did not accelerate functional recovery further following facial nerve injury [51]. A delayed axonal regeneration in *Grn* ko mice was also observed after sciatic nerve injury [61]. Notably, axonal regeneration, restoration of neuromuscular synapses and recovery of motor and sensory functions were significantly accelerated in transgenic mice overexpressing PGRN in neurons compared to normal control mice, and these effects were linked to the activation of Notch signaling pathways [61]. In another study, the same group used transgenic mice overexpressing PGRN in dorsal root ganglion neurons, and observed faster recovery from neuropathic pain and accelerated nerve healing following sciatic nerve injury. The beneficial effects of PGRN in this model were attributed to an improved autophagic flux [63]. Finally, exogenously administered PGRN has been shown to promote axonal regeneration of trigeminal ganglion cells and to accelerate the recovery of corneal sensitivity in a cornea injury model. Pharmacological inhibition of Wnt signaling abolished the pro-regenerative activity of PGRN [41].

Taken together, the present study is the first to demonstrate that PGRN promotes the survival and axonal regeneration of injured RGCs, thus identifying PGRN as a novel neurotrophic factor for these retinal projection neurons. Analyses of various disease models suggest that PGRN confers its neurotrophic effects through multiple mechanisms, including suppression of excessive neuroinflammation, improvement of lysosomal function, and activation of neurotrophic signaling cascades. How PGRN exerts the pro-survival and pro-regenerative effects on axotomized RGCs remains to be studied in future work. Furthermore, it will be interesting to analyze the impact of exogenously administered PGRN on the progression of RGC loss in animal models of clinically more relevant optic neuropathies, such as glaucoma.

## Data Availability

The raw data supporting the conclusions of this article will be made available by the authors on request.

## Supplementary Materials

The following supporting information can be downloaded at https://www.mdpi.com/article/10.3390/cells15110988/s1, Figure S1: Visualization of RGCs in retinal sections using BRN-3A immunohistochemistry.

## Abbreviations

The following abbreviations are used in this manuscript:

ANOVA	Analysis of variance
BDNF	Brain-derived neurotrophic factor
BRN-3A	Brain-specific homeobox/POU protein 3A
CAG	Cytomegalovirus enhancer/chicken β-actin
CLN11	Neuronal ceroid lipofuscinosis type 11
CNS	Central nervous system
CNTF	Ciliary neurotrophic factor
CTSD	Cathepsin D
DAPI	4′,6′-diamidino-2-phenylindole
dpl	Days post lesion
ERK	Extracellular signal-regulated kinase
FTLD	Frontotemporal lobar degeneration
GDNF	Glial cell line-derived neurotrophic factor
GRN	Granulin
IL-6	Interleukin-6
IOP	Intraocular pressure
kDa	Kilodalton
MAPK	Mitogen-activated protein kinase
MPTP	1-methyl-4-phenyl-1,2,3,6-tetrahydropyridine
NMDA	N-methyl-D-aspartate
NSCs	Neural stem cells
NTFs	Neurotrophic factors
P	Passage
PBS	Phosphate-buffered saline
PFA	Paraformaldehyde
PGRN	Progranulin
PI3K	Phosphatidylinositol-3-kinase
PNS	Peripheral nervous system
RGC	Retinal ganglion cell
rPGRN	Recombinant PGRN
SORT1	Sortilin
TDP-43	Transactivation response DNA-binding protein of 43 kDa
