# Supplementary material for: Progranulin Is a Survival Factor for Axotomized Retinal Ganglion Cells in Adult Mice
Source: Cells. 2026 May 28;15(11):988. doi: 10.3390/cells15110988 (PMC13256079; doi:10.3390/cells15110988)
Supplement: Supplementary file 1 [file cells-15-00988-s001.zip › cells-4286831-supplementary.pdf]

Supplementary Materials

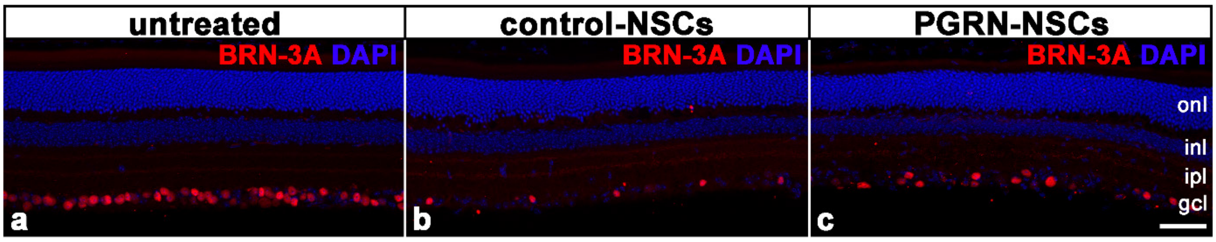

Figure S1. Visualization of RGCs in retinal sections using BRN-3A immunohistochemistry. Retinas treated with PGRN-NSCs (**c**) contained significantly more surviving RGCs 14 days after the lesion than retinas treated with control-NSCs (**b**). A retina of an animal that received neither an optic nerve crush nor an intravitreal cell transplant is shown for comparison **(a**). Abbreviations: BRN-3A, brain-specific homeobox/POU domain protein 3A; DAPI, 4',6-di-amidino-2-phenylindole; gcl, ganglion cell layer; inl, inner nuclear layer; ipl, inner plexiform layer; NSCs, neural stem cells; onl, outer nuclear layer; PGRN, progranulin; RGCs, retinal ganglion cells. Scale bar: 50 µm.
